# Systemic immune inflammation index is a valuable marker for predicting hemodialysis patients with depression: a cross-sectional study

**DOI:** 10.3389/fpsyt.2024.1423200

**Published:** 2024-08-05

**Authors:** Xi-xi Han, Hui-ying Zhang, Jing-wen Kong, Yu-xin Liu, Ke-ren Zhang, Wen-ying Ren

**Affiliations:** ^1^ Dongzhimen Hospital, Beijing University of Chinese Medicine, Beijing, China; ^2^ Nephrology Department, Beijing Integrated Traditional Chinese and Western Medicine Hospital, Beijing, China

**Keywords:** maintenance hemodialysis, depression, systemic immune-inflammation index, inflammatory marker, chronic inflammation

## Abstract

**Objective:**

Maintenance hemodialysis (MHD) patients suffer from enormous physical, mental stress and poor quality of life, so an increasing number of patients are in a long-term state of depression. A prominent feature of MHD patients is chronic persistent inflammation, which is also an important mechanism for the onset of depression. Therefore, finding economically convenient inflammatory markers to predict and diagnose the onset of depression in MHD patients is of great value. As a novel inflammatory marker, systemic immune inflammation index (SII) can more comprehensively reflect the inflammation and immunity level of patients. This study aims to explore the relationship between SII and depressive symptoms in MHD patients.

**Methods:**

A cross-sectional study was conducted on 206 MHD patients from three dialysis centers. Based on the Hospital Anxiety and Depression Scale (HADS) scores, patients were divided into non-depression and depression groups. Inter group comparison and multivariate logistic regression analysis were performed to determine whether SII is an independent risk factor for depression in MHD patients. Receiver operating characteristic (ROC) curve was used to evaluate the predictive value of SII on depression symptoms in MHD patients.

**Results:**

According to the HADS scale score, 38.83% of the included patients were in a state of depression. After adjusting for all confounding factors, MHD patients with SII>963.93 had a 4.709 times higher risk of depression than those with SII ≤ 478.32 (OR=4.709, 95% CI 1.821–12.178, P<0.01). ROC analysis showed that SII>685.11 was the best cutoff value for MHD depression patients, and the area under the curve (AUC) was 0.681.

**Conclusions:**

High SII is an independent risk factor for depressed MHD patients and an ideal inflammatory marker for predicting and identifying depression in MHD patients as assessed by the HADS scale.

## Introduction

Maintenance hemodialysis (MHD) remains a major method of renal replacement therapy for End Stage Kidney Disease (ESKD) patients, but it also brings many problems. Factors such as fatigue, pain, itching, sleep disorders, and cardiovascular disease (CVD) all cause immense physical and mental pressure on patients, leading to an increasing number of MHD patients experiencing anxiety and depression (1). According to statistics, compared to patients who have not undergone MHD, MHD patients have a 3–4 times higher prevalence of depression (2). A Korean cohort study showed that ESKD patients have a high risk of mental illness, with MHD patients having the highest prevalence of mental illness (3). Adverse psychological conditions can greatly reduce the compliance of MHD patients, thereby increasing hospitalization and mortality rates (4). Therefore, early identification of risk factors for depression in MHD patients is crucial.

Chronic inflammation is an important mechanism for the onset of depression, and a large number of studies have shown an increase in circulating immune cells such as pro-inflammatory cytokines, granulocytes, and macrophages in patients with depression, and it involves multiple inflammatory and immune pathways such as lipid peroxidation and oxidative stress (5, 6). Chronic kidney disease (CKD) patients, especially MHD patients, are often accompanied by chronic inflammation, which can induce many complications including depression, thereby reducing the quality of life of patients and increasing mortality rate (7). Due to the tendency of psychiatric disorders such as depression to be hidden, the gold standard for diagnosis is clinical interviews, which are easily overlooked by doctors. Therefore, it is necessary to use clinically stable and convenient indicators to identify and treat depression (8). The systemic immune inflammation index(SII), a biomarker, is an objective indicator of the balance between host systemic inflammation and immune response, taking into account the different immune and inflammatory response pathways involved by neutrophils, platelets, and lymphocytes (9). A high level of SII usually indicates an increased inflammatory response and decreased immune response in patients, which has been gradually discovered to be related to the onset and prognosis of various mental disorders in recent years (10). However, there have been no reports on the relationship between SII and depressive status in MHD patients. Therefore, this study investigated the correlation between SII and depression in MHD patients and evaluated the diagnostic value of SII for depressive status in MHD patients.

## Methods

### Patients and study design

This study is a cross-sectional study. We recruited 206 MHD patients who underwent regular dialysis at Beijing Hospital of Integrated Traditional Chinese and Western Medicine, Dongzhimen Hospital and Dongzhimen Hospital at Tongzhou Hemodialysis Center from February 2023 to December 2023.Individuals who underwent regular hemodialysis for at least 3 months and were able to cooperate with doctors to complete questionnaire surveys were included in this study. Individuals who underwent regular hemodialysis for at least 3 months and were able to cooperate with doctors to complete questionnaire surveys were included in this study. The exclusion criteria were: (1) having taken antidepressants within the past six months; (2) having a cold, fever, lung infection, or systemic infection within the past 2 weeks; (3) having taken non steroidal anti-inflammatory drugs, corticosteroids, immunosuppressive drugs, or antibiotics within the past 2 weeks; (4) having severe heart, brain, liver complications Pulmonary dysfunction, hematological disorders, malignant tumors. This study was approved by Ethics Committee of Dongzhimen Hospital Affiliated to Beijing University of Chinese Medicine (2024DZMEC-313-02). All research processes comply with Helsinki Declaration.

### Data collection

The sociodemographic data of patients were collected through interviews, and laboratory indicators were obtained by collecting fasting blood from patients before dialysis. Whole blood cell analysis and pre dialysis biochemistry were measured using a fully automated flow cytometer and a fully automated biochemical analyzer, respectively. The calculation formula for SII is: platelet count×Neutrophil count÷lymphocyte count.

### Evaluation of depressive symptom status

This study used the Hospital Anxiety and Depression Scale (HADS) to screen MHD patients in a depressed state, as the scale is specifically designed for individuals with illnesses and does not confuse physical symptoms caused by the disease. Many studies have confirmed that it is a good screening tool for depression and anxiety in the CKD population (11–13). This scale consists of 14 items, which are used for screening anxiety status (HADS-A) and depression status (HADS-D). Each item has four possible answer options (with four options ranging from 0 to 3 points). The total scores of subscales HADS-A and HADS-D are the sum of seven items, with scores ≥ 8 indicating anxiety and depression status.

### Statistical analysis

Analyze the data and draw using SPSS 26.0 (version 26.0; SPSS Inc., Chicago, Illinois) and GraphPad Prism 9.3.1 (GraphPad, San Diego, CA, USA). Quantitative data that conforms to a normal distribution are represented by Mean ± SD, those that do not conform to a normal distribution are represented by Median (Q1–Q3), and count data are represented by frequency (percent). The comparison of econometric data between two groups follows a normal distribution using independent sample t-test, while non normal distribution follows a Mann Whitney U-test, and count data follows a chi square test. Logistic regression analysis was used to investigate the correlation between depression status and SII levels in MHD patients. Model 1 did not adjust for any variables, which is equivalent to Univariate Analysis and Multivariate logistic regression analysis to determine whether SII is an independent risk factor for depression in MHD patients. This study used three models to control for consolidation and ultimately determine whether SII is an independent risk factor for depression. Receiver operating characteristic (ROC) curve analysis the diagnostic ability of SII and diagnostic cutoff for depressive symptoms in MHD patients. A p-value < 0.05 was considered statistically significant.

## Results

### Demographic characteristics

This study included 206 MHD patients, according to the HADS questionnaire assessment, 38.83% of hemodialysis patients are in a state of depression (n=80). The mean age of the depressed group was 59.88 ± 11.01 years, while the non-depressive group was 58.13 ± 13 years. The comparison of the demographic data between the two groups is shown in **Table 1**. Compared with non-depression group, the depression group had a higher BMI and lower years of education(p<0.05), but other variables have no statistical significance (p>0.05).

**Table 1:** Socio-demographic characteristics of MHD patients.

Variables	Depression(n=80)	Non-depression (n=126)	*P*
Age, years	59.88 ± 11.01	58.13 ± 13	0.305
Male, n (%)	48 (60%)	74 (58.73%)	0.857
BMI, kg/m^2^	24.25 ± 3.5	23 ± 3.66	0.016
Education, years	9 (9, 12)	12 (9, 15)	0.002
Dialysis time (months)	43.5 (14, 105)	36 (12.75, 84)	0.47
Smoking, n (%)	20 (25%)	27 (21.43%)	0.552
Alcohol consumption, n (%)	6 (7.5%)	18 (14.29)	0.139
Marital status, n (%)			0.943
Unmarried	3	7	
Married	67	104	
Divorced	3	4	
Widowed	7	11	
Monthly income, n (%)			0.089
≤3000	31	31	
3000–5000	13	22	
≥5000	36	73	
Primary disease, n (%)			0.35
Diabetic nephropathy	35	54	
Hypertensive nephropathy	27	37	
Glomerulonephritis	6	8	
IgA nephropathy	0	5	
MODS	1	0	
Other	11	22	

Values are presented as means ± SD or Median (Q1–Q3)/N (%)

BMI, body mass index; MODS, multiple organ dysfunction syndromes.

### The laboratory characteristics

In terms of laboratory indicators, the HGB, Neutrophil count, SII, and TG were significantly higher in the depression group than in the non-depression group, Lymphocyte count and Mg were significantly lower in the non-depression group than in the depression group (p<0.05), other indicators had no significant difference between the two groups (p>0.05), **Table 2** shows the laboratory characteristics of MHD patients.

**Table 2:** Laboratory characteristics of MHD patients.

Variables	Depression (n=80)	Non-depression (n=126)	*P*
WBC (×10^9^/L)	6.63 (5.48, 7.52)	6.13 (5.27, 7.12)	0.058
HGB (g/L)	119 (109, 127)	116 (105.75, 123)	0.033
NEUT# (×10^9^/L)	4.77 (3.96, 5.72)	4.06 (3.49, 5.09)	0.004
LYMPH# (×10^9^/L)	1.04 (0.83, 1.32)	1.2 (0.92, 1.5)	0.017
MONO# (×10^9^/L)	0.4 (0.35, 0.5)	0.4 (0.32, 0.5)	0.514
PLT (×10^9^/L)	177 (155, 224.75)	176 (141, 218.25)	0.423
SII Median (Q1–Q3)	843.92 (595.08, 1173.43)	605.73 (428.78, 844.96)	<0.0001
Scr (μmol/L)	913.31 ± 245.33	909.1 ± 254.21	0.906
BUN (mmol/L)	26.42 ± 6.65	27.55 ± 7.92	0.295
UA (μmol/L)	407.87 ± 96	421.36 ± 102.22	0.346
GLU (mmol/L)	7.66 (6.42, 11.5)	7.6 (6, 10.42)	0.433
ALP (U/L)	84 (65, 105.1)	86 (69.9, 115)	0.411
ALB (g/L)	39.6 (37.8, 42.1)	40 (37.35, 42.1)	0.954
PAB (mg/L)	318 (244, 383)	310 (247, 365.5)	0.585
TC (mmol/L)	3.47 (3.07, 4.22)	3.82 (3.08, 4.44)	0.133
TG (mmol/L)	1.91 (1.33, 3.14)	1.71 (1.04, 2.33)	0.031
Ca (mmol/L)	2.2 (2.09, 2.35)	2.22 (2.09, 2.35)	0.768
P (mmol/L)	1.88 ± 0.6	1.86 ± 0.54	0.824
Na (mmol/L)	138.5 (136.6, 139.7)	138.1 (135.8, 139.83)	0.363
CL (mmol/L)	100.35 ± 4.08	99.48 ± 3.91	0.127
K (mmol/L)	4.93 ± 0.66	5.11 ± 0.71	0.059
Mg (mmol/L)	1.06 ± 0.16	1.11 ± 0.18	0.048
CO_2_cp (mmol/L)	21.65 (20.2, 23.4)	21.9 (20, 23.7)	0.504
Fer (μg/L)	379.3 (234.38, 797.5)	330.3 (209.4, 687.76)	0.246
iPTH (pg/mL)	260.35 (147.3, 488.85)	268.3 (185.95, 438.75)	0.726

Values are presented as means ± SD or Median (Q1-Q3)/N (%)

WBC, white blood cells; HGB, hemoglobin; NEUT#, neutrophils; LYMPH#, lymphocytes; MONO#, monocytes; PLT, platelets; SII, systemic immune-inflammatory; Scr, serum creatinine; BUN, blood urea nitrogen; UA, uric acid; GLU, glucose; ALP, alkaline Phosphatase; ALB, albumin; PAB, prealbumin; TC, total cholesterol; TG, triglyceride; CO_2_cp, carbondioxide combining power; Fer, ferritin; iPTH, intact Parathyroid Hormone.

### Multivariate logistic regression analysis between SII and hemodialysis patients with depression

In order to better reveal that SII has an increasing trend for the depression incidence, this study represents SII as quartiles for logistic regression. The results of the multivariate logistic regression analysis are shown in **Table 3**. The crude model, model II and model III showed that there is a positive correlation between depression and SII in MHD patients. Model IV was adjusted for Age, Sex, BMI, Education, dialysis time, Marital status, Monthly income, Smoking, Alcohol consumption, Primary disease, HGB, Scr, BUN, UA, GLU, ALP, ALB, PAB, TC, TG, Ca, P, Na, CL, K, Mg, CO_2_cp, Fer, and iPTH. The results showed that MHD patients with SII>963.93 had a 4.709 times higher risk of depression than those with SII ≤ 478.32(OR=4.709, 95% CI 1.821–12.178, P<0.01).

**Table 3:** The odds ratio (95% CI) of depressed stratified by SII.

	OR (95%CI)				*P* for trend
	SII ≤ 478.32	478.32<SII≤686.82	686.82<SII≤963.93	SII>963.93	
Model 1	Reference	1.39 (0.575, 3.357)	2.444 (1.047, 5.707)	5.167 (2.196, 12.157)	0.00005
P		0.466	0.039	0.0002	
Model 2	Reference	1.327 (0.543, 3.242)	2.314 (0.981, 5.456)	5.019 (2.114, 11.917)	0.000088
P		0.535	0.055	0.000256	
Model 3	Reference	1.487 (0.586, 3.773)	2.537 (1.029, 6.254)	5.286 (2.128, 13.131)	0.000179
P		0.404	0.043	0.000335	
Model 4	Reference	1,206 (0.462, 3.152)	1.738 (0.683, 4.423)	4.709 (1.821, 12.178)	0.002
P		0.702	0.246	0.001	

OR, odds ratio.

Model 1: crude.

Model 2: adjusting for Age, Sex, BMI.

Model 3: adjusting for Age, Sex, BMI, Education, Dialysis time, Marital status, Monthly income, Smoking, Alcohol consumption, Primary disease.

Model 4: adjusting for Age, Sex, BMI, Education, Dialysis time, Marital status, Monthly income, Smoking, Alcohol consumption, Primary disease, HGB, Scr, BUN, UA, GLU, ALP, ALB, PAB, TC, TG, Ca, P, Na, CL, K, Mg, CO_2_cp, Fer, iPTH.

### ROC curve analysis

**Figure 1:**
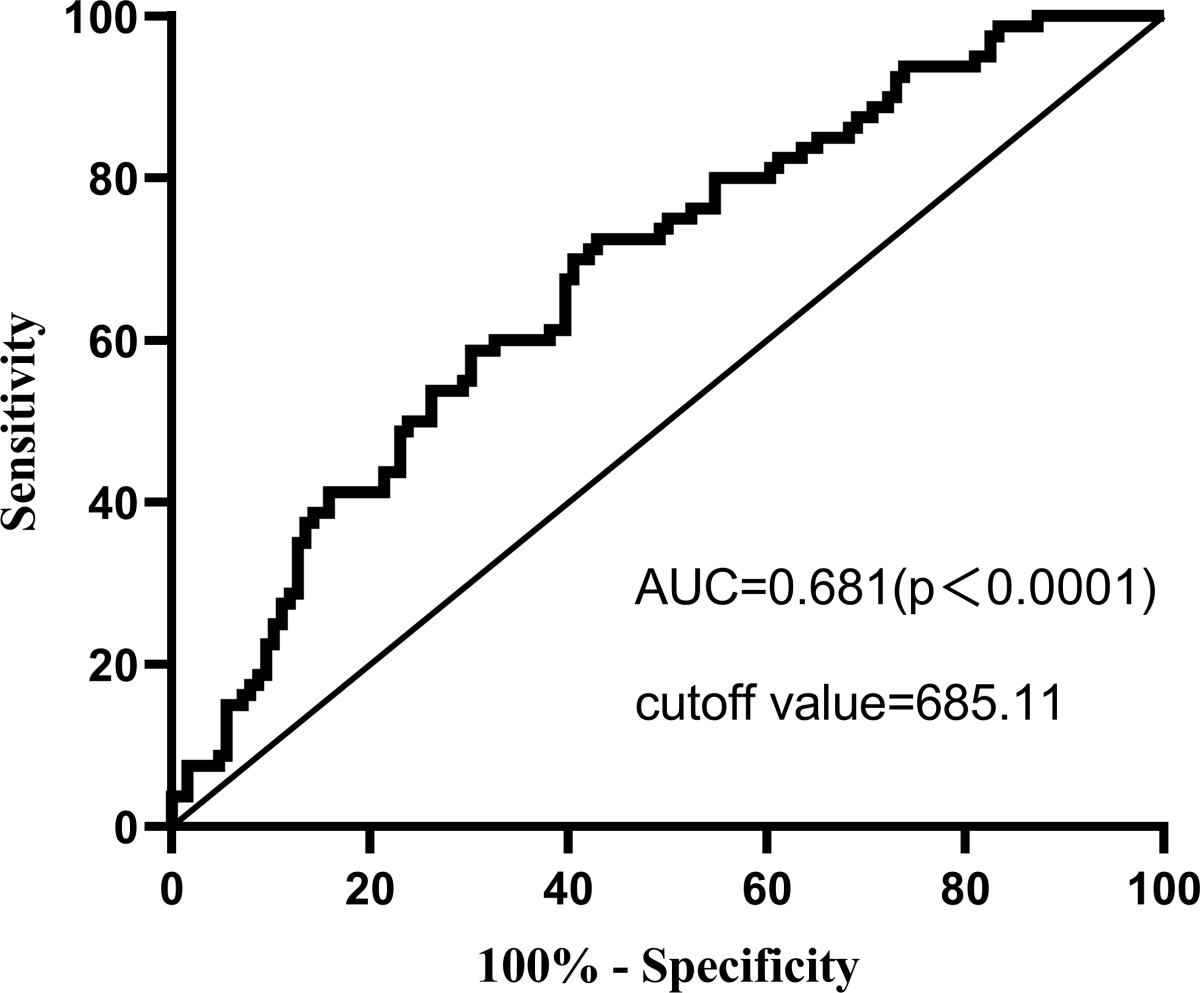
The ROC curve analysis of systemic immune-inflammation index.

As shown in **Figure 1**, The ROC indicates that the optimal cut-off for SII diagnosis of depression state (the HADS scale indicating the presence of depression) in MHD patients is 685.11, with a sensitivity of 0.725 and a specificity of 0.571. The area under the ROC curve was 0.681 (AUC=0.681, 95% CI=0.607, 0.754, P<0.0001).

## Discussion

The key results in this study is that SII was an independent risk factor in hemodialysis patients in a depressed state(according to the HADS scale), SII is an ideal inflammatory marker for diagnosing and predicting depressive symptoms in MHD patients.

Our research using HADS scale indicates that 38.83% of MHD patients are in a state of depression, similar to previous reports, Othayq et al. reported that 43.6% of MHD patients experience depression, the study used an anxiety and stress scale 42 to screen for depression symptoms in hemodialysis patients (14). Saulo B V de Alencar et al. using 5-item Geriatric Depression Scale reported that 43.3% of MHD patients have depressive symptoms (15). However, Ye et al. found that the prevalence of depression in MHD patients was 68.93% (Beck Depression Inventory) (16). This difference may be related to different assessment questionnaires, different economic and cultural levels and religious beliefs, but it can be determined that the incidence rate of depression in MHD patients is about 3–4 times higher than that in non MHD population (2).

Chronic persistent inflammation is one of the most prominent characteristics of uremic patients and a major factor leading to various complications including depression (7). MHD patients have an active response to foreign substances, while their normal immune response function is impaired. During dialysis, contact between blood and foreign objects can lead to acute and chronic inflammatory reactions through complement activation, cytokine or nitric oxide (NO) production, oxidant stress, carbonyl stress, activation of T-cell, B-cell and monocyte et al. (17). In addition, MHD patients can also experience sustained inflammatory activation due to factors such as oxidative stress, hypoxia, fluid and sodium overload, dysbiosis of gut microbiota, and uremic toxins including indoxyl sulphate (7, 18). High levels of inflammation in MHD patients can further mediate the occurrence of depression through cytokines. Pro-inflammatory cytokines can directly or indirectly stimulate the hypothalamic-pituitary-adrenal(HPA) axis and sympathetic nervous system(SNS) axis to overactivate indoleamine 2,3-dioxygenase(IDO), breaking down tryptophan into neurotoxic metabolites such as canine uric acid instead of 5-hydroxytryptamine(5-HT), leading to depression (19, 20). In addition, neuroinflammation also has toxic effects on the emotional regulation areas of the brain (21). The pathological mechanism of depression in inflammatory diseases such as uremia is mainly due to the entry of inflammatory cytokines into the central nervous system through bodily fluids and neural pathways, which then interact with each other, leading to inflammation and immune responses (22). Therefore, personalized anti-inflammatory treatment for depressed patients with chronic persistent inflammation has become an emerging and promising development direction (23).

The calculation formula for SII index is platelet count×Neutrophil absolute value÷lymphocyte absolute value, these three indicators are all obtained from routine blood tests, which are simple and convenient to obtain, and can comprehensively reflect the inflammatory and immune levels of the body. In recent years, more and more studies have shown their value in predicting poor prognosis of intracerebral hemorrhage, urological cancer, sepsis, identifying active ulcerative colitis, hepatic steatosis, psoriasis activation, and other diseases (24–29).

The correlation between SII and depression has also been reported in many studies. As one study suggests, SII is an independent risk factor for depression, with a 2% increase in the risk of depression for every 100 units of increase (30). Another cross-sectional study on patients with depression showed that high SII at admission can be used to identify moderate/major depression, indicating that early prevention of depression can be based on SII and anti-inflammatory treatment can be used to avoid major depression (31). The peripheral immune system involvement mechanism of depression is the activation of the innate immune system, represented by neutrophils, to produce cytokines, activate the complement system, and activate the adaptive immune system through antigen presentation (32). High SII represents an increase in neutrophils and platelets, a decrease in lymphocytes, indicating an enhanced inflammatory response and impaired immune system. Neutrophils can release pro-inflammatory cytokines and lead to oxidative stress, forming an inflammatory environment that promotes the progression of depression (33). Secondly, neutrophils release neutrophil elastase during the inflammatory process, increasing the levels of reactive oxygen in the body. Excessive production of reactive oxygen can lead to extensive protein and lipid peroxidation, increased blood-brain barrier permeability, and ultimately neuroinflammation, which is also an important mechanism for the formation of depression (34). Several studies have observed an increase in neutrophils in MDD patients and animals under social stress (35, 36). Lymphocytes are the main executors of almost all immune functions in the lymphatic system, and their involvement in depression is due to the reduction of T and B cells. T regulatory cells can maintain normal immune function (37). B cells can release anti-inflammatory factors to reduce harmful immune responses, and the reduction of both can create a pro-inflammatory environment, leading to the occurrence of acute depression (38). Animal experiments have also shown that naive lymphopenic Rag2 −/− mice exhibit less depression, decreased pro-inflammatory cytokines, and hippocampal cell proliferation after receiving lymphocyte transfer from mice that have experienced stress, indicating that adaptive immunity carried out by lymphocytes has anti stress and anti-depression effects (39). Platelet activation can produce pro-inflammatory cytokines and activating substances, initiating an inflammatory state to participate in the occurrence of depression (40). Therefore, SII can more comprehensively reflect the level of inflammation in the body, and thus more comprehensively and stably evaluate the state of depression.

Previous study has investigated the correlation between depression status and inflammation index in MHD patients, indicating that high NLR is an independent risk factor for depression scores in MHD patients and can be used to predict depression status in MHD patients (41). This study reports for the first time the correlation between SII and depressive status as assessed by the HADS scale in MHD patients, and the results show that high SII is an independent risk factor for depressive status in MHD patients. The risk of depression in MHD patients with SII>963.93 is 4.709 times that of those with SII ≤ 478.32, and SII>685.11 is the cutoff for diagnosing depression in patients. Similar to the results of this study, SII has also been shown to be an independent risk factor for depression in other chronic inflammatory diseases, Liu et al. found through logistic regression analysis that SII is significantly correlated with anxiety and depression in pulmonary tuberculosis patients (42). Wang et al. showed that high SII level was an independent risk factor for depression in diabetes patients through multivariate logistic regression analysis. Inflammation is an important pathogenesis of stroke, and its inflammatory storm is also involved in the onset of post-stroke depression (43). The level of SII at admission is significantly correlated with the occurrence of depression one month later, and logistic regression shows that SII>547.30 is significantly correlated with post-stroke depression(OR=2.181,95% CI=1.274–3.732, p=0.004) (44). COVID-19 can also lead to persistent inflammation, and SII is positively correlated with anxiety and depression scores at one month follow-up in COVID-19 patients, which can serve as a biomarker for diagnosing and treating psychological problems related to COVID-19 (45, 46).

This study has some limitations. Firstly, this is a cross-sectional study, the causal relationship cannot be obtained. In the future, prospective studies should be conducted to further analyze the causal relationship between the inflammation and depression in MHD patients. Secondly, this study only investigated three dialysis centers, and some patients who refused to be interviewed during the survey would result in a limited number of included patients. In the future, the scope of the survey should be expanded nationwide to evaluate the depression status and inflammation index of hemodialysis patients. Thirdly, we only used the HADS scale instead of interviews with psychiatrists to diagnose the depression of MHD patients. Although our researchers strictly evaluated MHD patients according to the questionnaire, the HADS scale is only a screening tool. So in future research and clinical work, psychiatrists should be involved in the management of hemodialysis patients, so as to better diagnose and treat patients’ depression and ultimately reduce the incidence rate of depression.

## Conclusion

In conclusion, this study demonstrates a positive correlation between SII and depressive symptoms in patients with MHD as assessed by the HADS scale, and high SII is an independent risk factor for depression in MHD patients. SII, as a new inflammatory biomarker that is easily accessible and economically convenient for dialysis centers, can provide certain reference for predicting and treating depression in MHD patients.

## Data availability statement

The original contributions presented in the study are included in the article/**Supplementary Material**. Further inquiries can be directed to the corresponding author.

## Ethics statement

The studies involving humans were approved by Ethics Committee of Dongzhimen Hospital Affiliated to Beijing University of Chinese Medicine approved this study (2024DZMEC-313-02). The studies were conducted in accordance with the local legislation and institutional requirements. The participants provided their written informed consent to participate in this study.

## Author contributions

X-xH: Data curation, Writing – original draft, Writing – review & editing. H-yZ: Data curation, Methodology, Writing – original draft. J-wK: Methodology, Writing – original draft. Y-xL: Data curation, Writing – original draft. K-rZ: Supervision, Writing – original draft. W-yR: Project administration, Supervision, Writing – review & editing.
